# Temporal changes of the life and renal prognoses of patients with rapidly progressive glomerulonephritis in Japan, 1989–2019

**DOI:** 10.1007/s10157-025-02643-6

**Published:** 2025-03-25

**Authors:** Kentaro Nakajima, Shuzo Kaneko, Joichi Usui, Naotake Tsuboi, Hitoshi Sugiyama, Shoichi Maruyama, Yoshitaka Isaka, Ichiei Narita, Kunihiro Yamagata

**Affiliations:** 1 Department of Nephrology, Institute of Medicine, University of Tsukuba, 1-1-1 Tennodai, Tsukuba, Ibaraki 305-8575 Japan; 2 Graduate School of Comprehensive Human Sciences, University of Tsukuba, Tsukuba, Japan; 3 Department of Nephrology, Itabashi Chuo Medical Center, Tokyo, Japan; 4 Department of Nephrology, School of Medicine, Fujita Health University, Toyoake, Japan; 5 Department of Medical Care Work, Kawasaki College of Allied Health Professions, Okayama, Japan; 6 Department of Nephrology, Internal Medicine, Nagoya University Graduate School of Medicine, Nagoya, Japan; 7 Department of Nephrology, Graduate School of Medicine, Osaka University, Suita, Japan; 8 Division of Clinical Nephrology and Rheumatology, Niigata University Graduate School of Medical and Dental Sciences, Niigata, Japan

**Keywords:** Rapidly progressive glomerulonephritis, Anti-neutrophil cytoplasmic antibody-associated vasculitis, Anti-glomerular basement disease, Questionnaire survey

## Abstract

**Background:**

This study is a continuation of the Japan Rapidly Progressive GlomeruloNephritis (RPGN) Working Group’s chronological nationwide survey.

**Methods:**

We analyzed 1,660 RPGN cases from 2016–2019 and compared them to 4,179 cases from five earlier periods (1989–1998, 1999–2001, 2002–2008, 2009–2011, 2012–2015). Data on causative diseases, clinical severity, 24-month life and renal survival, and treatment details were collected and compared.

**Results:**

The most recent cohort showed an older median age at onset (median age 74 years), with improved serum creatinine levels (median 2.5 mg/dL). Cumulative survival at 24 months remained stable (periods 1989–1998, 1999–2001, 2002–2008, 2009–2011, 2012–2015, 2016–2019 were each 72.0%, 72.9%, 77.7%, 83.0%, 84.9%, 83.5%, *p* < 0.01), while renal survival showed a favorable trend in the most recent periods (there were each 68.7%, 75.4%, 76.7%, 73.4%, 78.2%, 78.4%, *p* < 0.01). Anti-neutrophil cytoplasmic antibody-associated vasculitis (AAV)-RPGN had similar outcomes to the overall cohort. Increased rituximab use was observed, with no significant differences in life and renal prognosis between rituximab (RIX) and cyclophosphamide (CY). In severe renal impairment (Cre ≥ 6), renal prognosis was better in the CY or RIX use group than in the non-use group (*p* = 0.035, 0.025). Anti-glomerular basement membrane disease had a poorer renal prognosis compared to other causes.

**Conclusions:**

Despite an increasingly older age of onset, both life and renal prognoses for new-onset AAV-RPGN from 2016 to 2019 remain comparable to the best in previous surveys, due to the impact of constant improvements in early diagnosis and changes in treatment.

**Supplementary Information:**

The online version contains supplementary material available at 10.1007/s10157-025-02643-6.

## Introduction

Rapidly progressive glomerulonephritis (RPGN) is a severe clinical syndrome that can result in a severe decline in an individual’s renal function. If untreated, RPGN can quickly progress to end-stage kidney disease. Although the etiology of RPGN is diverse, the typical renal pathological features are crescentic glomerulonephritis or glomerulonephritis with crescent formation, and these lesions can present as renal limited vasculitis or partial systemic vasculitis. The most common cause of RPGN is anti-neutrophil cytoplasmic antibody (ANCA)-associated vasculitis (AAV) with pauci-immune type immunostaining. The cause of the most severe renal outcome form of RPGN is anti-glomerular basement membrane (GBM) disease (aGBMD) [1].

To date, the RPGN Working Group of the Research Committee of Intractable Renal Disease has been conducting continuous surveys to follow clinical RPGN practices in Japan [1–5]. This nationwide survey has been conducted retrospectively at intervals for the facilities in Japan with nephrologists, and a total of 4179 new-onset RPGN cases identified during the years 1989 to 2015 have been recorded in these surveys. Although the overall life expectancy of individuals with RPGN has improved, their renal prognosis has not improved since 1999 [1–5]. In Japan, the age of onset of infection is older, and the number of infection-related deaths has increased in elderly patients due to concomitant use of immunosuppressive drugs such as cyclophosphamide (CY), and the improvement in renal prognosis with CY is limited in elderly patients [2]. Thus, Japanese guidelines have recommended glucocorticoid (GC) monotherapy as an option to prevent infection-related deaths has contributed to the improvement. Due to differences in patient backgrounds between Japan and western countries, the majority of patients in Japan with AAV-RPGN are treated with GC monotherapy, indicating a gap between the evidence obtained from the clinical trials in western countries and the actual status of the treatment in Japan [1, 6–8]. CY has traditionally been used as an immunosuppressive agent for the initial therapy for RPGN, but the newly reported usefulness of rituximab (RIX) [9, 10] has made this monoclonal antibody a new option for initial therapy. In the present study, we conducted the latest survey of new-onset RPGN cases during the period 2016–2019 in Japan as a continuation of the nationwide survey, and we report the differences in life and renal prognoses that depend on the period of disease onset or other prognostic determinants and changes in treatment.

## Patients and methods

The nationwide survey is a multicenter retrospective observational analysis of patients’ response data, without the collection of samples. Prior to the survey, a brief questionnaire was sent to registered nephrologist facilities in Japan as a preliminary survey to estimate the number of new-onset RPGN patients identified between April 2016 and March 2020: 4651 responses from 429 facilities were identified. The survey was mailed to each facility in November 2022, and a total of 1840 cases (39.6%) was obtained. It could not be determined whether or not 180 cases had developed during the current survey period, and these cases were excluded, leaving a final total of 1,660 cases for analysis. We used the total of 4179 RPGN cases recorded in the same survey in 1989–2015 for comparison with the present study’s data.

The breakdown of the data is as follows: 884 cases from 1989 to 1998 (period ‘89–’98), 321 cases from 1999 to 2001 (period ‘99–’01), 567 cases from 2002 to 2008 (period ‘02–’08), 1,021 cases from 2009 to 2011(period ‘09–’11), and 1,386 cases from 2012 to 2015 (period ‘12–’15). The time period for the present survey was 2016–2019 (period ‘16–’19). The present survey’s items were as follows: age at onset, gender, causative disease, lung lesion(s) (presence/absence), serum creatinine (Cre) level at diagnosis, day of starting initial treatment, serum C-reactive protein (CRP) level, serum myeloperoxidase (MPO)-ANCA level, proteinase (PR)3-ANCA level, anti-glomerular basement membrane (GBM) antibody level, the content of the patient’s initial/maintenance treatment (GC, immunosuppressive drugs, CY, RIX; apheresis [use/non-use; modality], renal outcome on the final follow-up day [dialysis dependent/independent or transient]), life outcome on the final follow-up day (alive/dead), and cause of death.

The clinical severity (CS) at onset grade was also recorded. The CS grade was defined in accord with the first clinical guideline for RPGN in Japan (2002) [11], based on the patients’ age, Cre, CRP, and the presence/absence of lung lesion(s); we also stratified the CS grades by the sum of each score for predicting the life prognosis and the renal prognosis. The CS grades were defined as follows: Age: < 60 years = 0, 60–70 years = 1 point, > 70 years = 2 points. Serum Cre level: < 3 mg/dL = 0 points, 3 to < 6 mg/dL = 1 point, ≥ 6 mg/dL = 2 points, dialysis dependent at diagnosis = 3 points. Serum CRP level: < 2.6 mg/dL = 0 points, 2.6–10 = 1 point, > 10 mg/dL = 2 points. Lung lesion(s): yes = 2 points, no = 0 points. Total score (Age + Cre + CRP + Lung lesion): 0–2 points = CS grade I, 3–5 points = CS grade II, 6–7 points = CS grade III, 8–9 points = CS grade IV.

### Statistical analyses

Between-group comparisons were conducted for each pair of groups. For the comparisons of score data or continuous data, we used an unpaired *t*-test for the mean differences in data that followed a normal distribution, and we used a non-parametric analysis of median differences (Mann–Whitney *U*-test) otherwise. The χ^2^-test or Fisher’s exact test was used for comparisons of categorical data such as frequencies. The results of the comparisons of patient survival and renal-cumulative survival are presented as the Kaplan–Meier curve with the log-rank test. We used the log-rank trend test and the Jonckheere–Terpstra trend test to examine trends. A renal survival analysis was performed using the death-censored analyses. All presented p-values were two-sided tests, and *p*-values < 0.05 were accepted as significant. The statistical analyses were performed with SPSS Statistics ver. 29.0. 2.0 (20) for Windows (IBM).

### Ethics approval and consent to participate

The study protocol was approved by the Ethics Review Board of the University of Tsukuba Hospital (No. R04-158, 27th October 2022), and the study complied with the Declaration of Helsinki. The requirement for patients’ informed consent was waived because all data included in this dataset were de-identified.

## Results

### Changes in causative disease over time

**Fig. 1:**
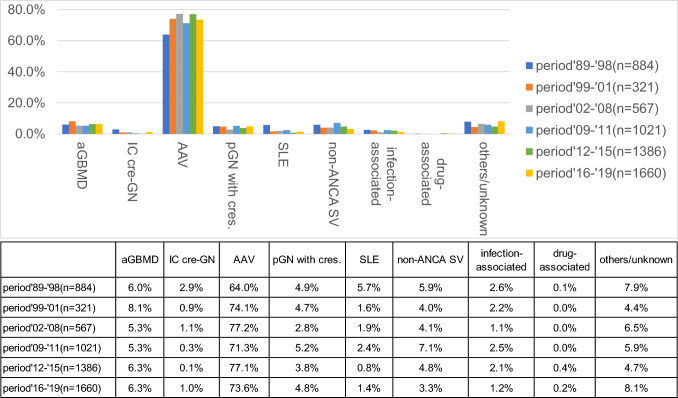
The causative diseases of rapidly progressive glomerulonephritis (RPGN) from the nationwide survey in Japan from 1989 to 2019. *aGBMD* anti-GBM nephritis, Goodpasture syndrome. AAV (anti-neutrophil cytoplasmic antibody [ANCA]-associated vasculitis): MPA (microscopic polyangiitis), RLV (renal limited vasculitis), GPA (granulomatosis with polyangiitis), EGPA (eosinophilic granulomatosis with polyangiitis), unclassified ANCA-positive GN (glomerulonephritis), MPO/PR3-ANCA-double negative/unknown-pauci-immune crescentic GN. pGN with cres (primary glomerulonephritis with crescent): membranoproliferative GN, membranous nephropathy, IgA/non-IgA mesangial proliferative GN, and other GN with cres. Non-ANCA SV IgA vasculitis, cryoglobulinemic vasculitis, rheumatoid vasculitis, and malignancy-related vasculitis. Infection associated: post-streptococcal GN, infectious endocarditis, shunt nephritis, sepsis, abscess, hepatitis B virus infection, hepatitis C virus infection, and other infections. Others/unknown: unclassified/unknown/no available data. *aGBMD* anti-glomerular basement membrane (GBM) disease, *IC-cres GN* immune complex-type crescentic glomerulonephritis, *non-ANCA SV* non-anti-neutrophil cytoplasmic antibody (ANCA) systemic vasculitis, *n* number, *SLE* systemic lupus erythematosus

Figure 1 depicts the percentages of the causative diseases in each period. In this study, we classified the causative diseases of the patients according to the clinical diagnosis obtained in previous studies. AAV was defined based on the same definition of AAV as that used in the previous surveys; patients with pauci-immune-type crescentic glomerulonephritis with negative or unknown MPO-ANCA or PR3-ANCA antibody titers were classified as having AAV. The results showed that AAV was the most common disease group in all groups, accounting for 73.6% of the cases in the period ‘16–’19.

### Changes over time in clinical parameters and the CS grade at onset

Table 1 summarizes the data on patient age, gender, Cre and CRP levels, presence/absence of lung involvement, and the prevalence of each CS grade over the six time periods. The patients’ mean and median age at onset persistently increased in the group of AAV patients and in the overall RPGN series. The Cre levels at onset showed a decreasing trend compared to the earlier five time periods. The CS scores tended to increase in both the overall RPGN and AAV-RPGN groups but did not differ significantly between the periods ‘02–’08 and ‘12–’15 in the RPGN series as a whole and period ‘12–’15 in the AAV patients as a whole. The CS grade was not significantly different between overall RPGN as a whole in period ‘12–’15 and AAV-RPGN as a whole in ‘09–’11 and later.

**Table 1:** Clinical characterisitics at onset

	Age		sCre(mg/dL)		CRP(mg/dL)		Lung involvement	Clinical severity classification	
	Mean ± SD	Median, range	Mean ± SD	Median, range	Mean ± SD	Median, range	%	Total score	CS Grade I/II/III/IV(%)
*antiGBMD*									
Period’89–’98 (*n* = 53)	52.66 ± 16.58^c−f^	56, 10–79	8.04 ± 4.90	7.0. 1.0–19.5	8.58 ± 7.72^f^	5.6, 0–26.0	21.2	3.81 ± 1.93^e,f^	26.4/58.5/11.3/3.8^d,e^
Period’99–’01 (*n* = 24)	57.13 ± 17.81^f^	61, 19–83	7.15 ± 4.25	6.2, 1.2–15.0	10.99 ± 10.07	8.7, 0.2–32.0	26.1	4.12 ± 1.99	29.2/37.5/33.3/0
Period’02–’08 (*n* = 30)	63.70 ± 17.35^a^	69, 11–93	6.30 ± 3.25	6.9, 0.4–13.2	12.61 ± 7.88	12.61, 0.4–29.5	33.3^f^	4.47 ± 2.57	30.0/33.3/23.3/13.4^d^
Period’09–’11 (*n* = 54)	65.94 ± 14.16^a^	70, 30–85	8.56 ± 5.76^f^	7.9, 1.9–28.9	11.95 ± 8.74	10.20, 0.2–45.5	19.6	4.76 ± 1.54	9.3/57.4/31.4/1.9^a,c^
Period’12–’15 (*n* = 88)	63.66 ± 16.82^a^	67, 10–90	8.17 ± 5.20^f^	7.1, 1.1–24.9	13.12 ± 9.22^f^	14.8, 0.14–35.2	27.3	5.09 ± 2.09^a,f^	11.4/47.7/30.7/10.2^a^
Period’16–’19 (*n* = 105)	64.67 ± 16.10^a,b^	69, 20–88	6.81 ± 5.25^d,e^	5.58, 0.53–31.99	11.27 ± 8.62^a,e^	10.7, 0.01–35.25	19.0^c^	4.53 ± 1.93^a,e^	17.1/50.5/25.7/5.7
*Overall AAV*									
Period’89–’98 (*n* = 565)	61.80 ± 14.81^b−f^	65, 6–86	4.99 ± 3.09^b−f^	4.3, 0.6–18.0	6.83 ± 7.08^e^	4.4, 0.1–45.0	27.7^b−f^	3.34 ± 2.07^c−f^	37.2/46.3/14.5/2.0^c−f^
Period’99–’01 (*n* = 236)	64.13 ± 15.35^d−f^	67.5, 5–91	4.30 ± 2.90^a,e,f^	3.7, 0.5–23.0	5.32 ± 5.33	3.8, 0.1–33.6	40.8^a,c,f^	3.49 ± 1.97^f^	33.9/48.9/15.1/2.1^d−f^
Period’02–’08 (*n* = 430)	67.37 ± 12.65^a,d−f^	70, 3–92	4.17 ± 2.55^a,e,f^	3.7, 0.2–16.2	6.22 ± 6.31	4.2, 0.1–33.2	49.1^a,b,d,e^	3.88 ± 1.81^a^	25.5/58.8/14.1/1.6^a,f^
Period’09–’11 (*n* = 722)	69.89 ± 13.13^a−c,f^	72, 3–97	3.90 ± 2.96^a,e,f^	3.2, 0.3–26.7	6.03 ± 6.53	3.8, 0–57.0	46.7^a.f^	3.85 ± 1.91^a^	24.6/55.2/17.5/2.7^a,b^
Period’12–’15 (*n* = 1066)	70.80 ± 13.74^a−c,f^	73, 3–94	3.38 ± 2.82^a−d,f^	2.50, 0.26–31.7	5.92 ± 6.53^a,f^	3.5, 0–36.0	42.9^a,c,f^	3.84 ± 1.95^a^	24.1/56.5/15.8/3.6^a,b^
Period’16–’19 (*n* = 1221)	72.46 ± 11.05^a−e^	74, 13–96	3.11 ± 2.62^a−e^	2.28, 0.20–25.7	6.72 ± 6.74^e^	4.68, 0–33.0	46.2^a,b,d,e^	3.95 ± 1.87^a,b^	21.8/53.6/18.9/2.5^a−c^
*MPA(+ RLV)*									
Period’89–’98 (*n* = 502)	62.57 ± 14.32^b−f^	65, 6–86	5.04 ± 3.09^b−f^	4.4, 0.6–18.0	6.82 ± 6.69^e^	4.5, 0.1–43.4	27.6^b−f^	3.39 ± 2.08^c−f^	36.9/45.9/15.0/2.2^c−f^
Period’99–’01 (*n* = 209)	65.01 ± 14.67^a,d−f^	68, 5–91	4.25 ± 2.89^a,e,f^	3.7, 0.5–23.0	5.24 ± 5.07	3.7, 0.1–24.2	39.3^a,c,f^	3.49 ± 1.97^f^	33.5/49.8/14.8/2.0^d−f^
Period’02–’08 (*n* = 378)	67.96 ± 12.33^a,d−f^	70, 3–92	4.16 ± 2.45^a,e,f^	3.7, 0.2–13.9	6.03 ± 6.05	3.9, 0.1–33.2	49.0^a,b,d,e^	3.89 ± 1.76^a^	24.6/61.1/12.7/1.6^a,f^
Period’09–’11 (*n* = 620)	70.27 ± 13.34^a−c,f^	73, 3–97	3.87 ± 2.98^a,e,f^	3.2, 0.3–26.7	5.84 ± 6.50^f^	3.5, 0–57.0	42.7^a,c,f^	3.83 ± 1.92^a^	25.0/55.0/17.3/2.7^a,b^
Period’12–’15 (*n* = 941)	70.85 ± 13.80^a−c,f^	73, 3–94	3.43 ± 2.86^a−d,f^	2.6, 0.26–31.7	5.89 ± 6.51^a,f^	3.4, 0–36.0	42.2^a,c,f^	3.85 ± 1.96^a^	24.4/56.1/16.0/3.6^a,b^
Period’16–’19 (*n* = 1165)	72.69 ± 10.93^a−e^	74.5, 13–96	3.11 ± 2.62^a−e^	2.31, 0.2–25.7	6.62 ± 6.68^d,e^	4.6, 0–33.0	46.2^a,b,d,e^	3.95 ± 1.86^a,b^	20.8/54.2/19.2/2.6^a−c^
*GPA*									
Period’89–’98 (*n* = 23)	46.68 ± 17.77^e−f^	49, 16–85	4.68 ± 3.41^e,f^	3.6, 0.7–13.0	8.77 ± 10.31	5.9, 0.1–42.5	34.8^d,f^	3.05 ± 1.84^d^	27.3/63.6/9.1/0^f^
Period’99–’01 (*n* = 9)	57.11 ± 12.89^e,f^	67, 5–91	5.16 ± 4.09^e^	5.3, 0.7–13.3	7.13 ± 3.72	8.0, 1.8–12.6	55.6	4.33 ± 2.60	22.2/44.4/22.2/11.1
Period’02–’08 (*n* = 14)	55.71 ± 18.89^e,f^	58, 14–80	3.91 ± 2.70	4.0, 0.6–8.3	8.77 ± 7.59	7.7, 0.7–24.5	69.2	4.07 ± 2.13	35.7/28.6/35.7/0^e^
Period’09–’11 (*n* = 19)	64.32 ± 16.81^a^	68, 5–79	3.66 ± 2.42^e^	3.1, 0.7–8.7	8.79 ± 7.21	7.4, 0–19.4	78.9^a^	4.84 ± 1.50^a,e^	10.5/57.9/31.6/0
Period’12–’15 (*n* = 35)	69.65 ± 12.62^a−c^	72, 21–86	2.30 ± 1.88^a,b,d^	1.6, 0.43–6.8	6.45 ± 7.06	4.7, 0–29.1	57.1	3.80 ± 1.89^d^	17.1/68.6/8.6/5.7^c^
Period’16–’19 (*n* = 43)	68.81 ± 12.27^a−c^	71, 39–87	3.00 ± 2.61^a^	2.08, 0.6–13.41	8.19 ± 6.67	8.46, 0.1–23.4	53.5^a^	4.12 ± 2.00	25.6/48.8/20.9/4.7^a^
*Primary GN with crescents*									
Period’89–’98 (*n* = 43)	46.09 ± 21.21^d,f^	55, 6–75	3.81 ± 3.25^f^	2.5, 0.8–16.2	2.76 ± 4.58	1.2, 0.1–21.3	2.4	1.47 ± 1.42	72.1/27.9/0/0^b^
Period’99–’01 (*n* = 15)	54.00 ± 21.69	63, 10–78	5.30 ± 3.86^e,f^	3.8, 1.5–14.3	3.13 ± 7.78	0.9, 0.2–28.9	0^e^	2.40 ± 2.10	66.7/20.0/13.3/0^a,d^
Period’02–’08 (*n* = 16)	50.94 ± 27.82	64, 3–78	2.90 ± 2.14	2.4, 0.2–8.8	1.17 ± 1.47	0.6, 0.1–5.8	0^e^	1.38 ± 1.15	81.3/18.8/0/0
Period’09–’11 (*n* = 53)	61.91 ± 17.39^a^	63, 9–91	2.66 ± 1.48	2.3, 0.5–7.5	1.08 ± 2.00	0.3, 0–8.7	3.8	1.64 ± 1.36	77.4/22.6/0/0^b^
Period’12–’15 (*n* = 53)	57.83 ± 16.39	60, 12–85	2.33 ± 1.44^b^	2.0, 0.5–8.5	1.87 ± 4.24	0.4, 0–23.9	7.5	1.32 ± 1.44	84.9/13.2/1.9/0
Period’16–’19 (*n* = 80)	60.71 ± 17.06^a^	63, 16–88	2.51 ± 1.66^a,b^	2.02, 0.65–7.30	2.28 ± 3.94	0.31, 0.01–15.60	8.8	1.80 ± 1.52	65.0/23.75/2.5/0
*SLE*									
Period’89–’98 (*n* = 50)	35.84 ± 14.70^d−f^	34, 13–72	2.96 ± 2.12	2.1, 0.9–13.0	1.94 ± 3.94	0.4, 0.1–15.6	12.8^b^	0.98 ± 1.13^f^	90.0/10.0/0/0^b,f^
Period’99–’01 (*n* = 5)	55.8 ± 12.34	56, 44–75	4.52 ± 3.18	3.5, 1.0–9.0	2.96 ± 2.00	1.8, 0.4–6.5	40^a,c^	2.60 ± 1.14	40.0/60.0/0/0^a,e^
Period’02–’08 (*n* = 11)	46.73 ± 20.0	47, 15–75	2.96 ± 2.00	2.0, 1.4–7.7	2.48 ± 2.54	2.2, 0.1–7.6	9.1^b^	1.82 ± 1.72	63.6/36.4/0/0
Period’09–’11 (*n* = 25)	48.88 ± 19.95^a,e^	52, 15–79	3.33 ± 2.59	2.6, 1.0–12.0	1.75 ± 4.04	0.5, 0–19.3	22.7	1.72 ± 2.15	64.0/28.0/4.0/4.0
Period’12–’15 (*n* = 11)	55.09 ± 17.71^a,d^	46, 34–86	2.74 ± 1.93	2.0, 0.7–6.7	4.54 ± 7.74	2.7, 0.1–27.0	18.2	2.45 ± 2.88	74.4/21.6/2.0/2.0^b^
Period’16–’19 (*n* = 24)	49.04 ± 20.41^a^	44.5, 15–81	2.62 ± 1.81	2.195, 0.48–9.18	2.51 ± 3.51	1, 0.02–15.21	25.0	1.92 ± 1.50^a^	62.5/37.5/0/0^a^
*Non-ANCA SV*									
Period’89–’98 (*n* = 52)	48.29 ± 20.80	53, 3–77	4.11 ± 2.88	3.5, 0.7–11.4	4.98 ± 6.96	1.8, 0.1–31.9	8.8	2.02 ± 1.86	69.2/25.0/5.8/0
Period’99–’01 (*n* = 13)	55.69 ± 15.93	58, 16–79	3.74 ± 2.40	2.9, 1.3–7.6	3.85 ± 4.76	0.8, 0.2–12.3	20	2.08 ± 1.55	53.8/46.2/0/0
Period’02–’08 (*n* = 23)	56.82 ± 24.89	68, 5–82	4.77 ± 3.55	3.1, 0.5–12.6	2.43 ± 3.63	1.1, 0.1–14.3	27.3	2.91 ± 2.49	54.5/27.3/13.6/4.5
Period’09–’11 (*n* = 72)	67.88 ± 12.71	71, 27–87	3.0 ± 1.83	2.4, 0.5–8.4	2.78 ± 4.26	1.39, 0–28.4	25.9	2.53 ± 1.46	54.2/43.1/2.8/0
Period’12–’15 (*n* = 66)	67.71 ± 13.55	70, 32–91	3.05 ± 1.98	2.3, 0.6–8.8	3.35 ± 4.65	1.4, 0–26.3	19.7	2.82 ± 1.74	48.5/45.5/4.5/1.5
Period’16–’19 (*n* = 54)	64.02 ± 20.59	70, 16–91	2.80 ± 2.91	1.86, 0.33–17.85	3.90 ± 4.64	1.965, 0.01–23.84	14.8	2.47 ± 1.90	53.7/37.0/5.6/1.9
*Infection-associated*									
Period’89–’98 (*n* = 52)	48.29 ± 20.80^d−f^	53, 3–77	4.12 ± 2.88	3.5, 0.7–11.4	4.98 ± 6.96	1.8, 0.1–31.9	8.7^c^	2.22 ± 1.41	56.5/43.5/0/0
Period’99–’01 (*n* = 13)	55.69 ± 15.93	58, 16–79	3.74 ± 2.40	2.9, 1.3–7.6	3.85 ± 4.76	3.3, 0.1–33.2	0^c^	2.43 ± 1.27	57.1/42.9/0/0
Period’02–’08 (*n* = 23)	56.82 ± 24.89	68, 5–82	4.77 ± 3.55	3.1, 0.5–12.6	2.42 ± 3.63	1.1, 0.1–14.3	71.4^a,b,d−f^	2.83 ± 1.72	66.7/33.3/0/0^d^
Period’09–’11 (*n* = 72)	67.88 ± 12.71^a^	71, 27–87	3.03 ± 1.83	2.4, 0.5–8.4	2.78 ± 4.26	1.1, 0–28.4	12.5^c^	2.42 ± 1.72	50.0/46.2/3.8/0^c^
Period’12–’15 (*n* = 66)	67.71 ± 13.55^a^	70, 32–91	3.05 ± 1.98	2.3, 0.6–8.8	3.35 ± 4.65	1.4, 0–26.3	6.9^c^	2.59 ± 1.59	55.2/37.9/6.9/0
Period’16–’19 (*n* = 20)	67.3 ± 16.50^a^	72.5, 27–88	3.98 ± 1.96	4.1, 0.92–8.66	5.65 ± 4.29	5.4, 0.71–16.65	10.0^c^	3.25 ± 1.55	40/55/5/0
*Total of overall RPGN*									
Period’89–’98 (*n* = 884)	57.33 ± 18.00^b−f^	63, 2–88	4.91 ± 3.34^d−f^	4.1, 0.3–19.5	6.15 ± 6.91	3.24, 0–45.0	22.7^b−f^	2.91 ± 2.10^a−d,f^	46.6/40.8/10.9/1.7^b−f^
Period’99–’01 (*n* = 321)	62.5 ± 16.05^a,c−f^	66, 5–91	4.73 ± 3.40^d−f^	3.9, 0.5–23.0	5.63 ± 6.22	3.60, 1–33.6	35.7^a,c,f^	3.42 ± 2.01^a,f^	36.0/46.8/15.6/1.6^a,e,f^
Period’02–’08 (*n* = 567)	64.79 ± 16.10^a,b,d−f^	69, 2–93	4.21 ± 2.66^e,f^	3.7, 0.2–16.2	6.00 ± 6.45	3.30, 0.1–33.2	44.0^a,b,d−f^	3.66 ± 2.00^a^	31.6/52.5/13.5/2.3^a,f^
Period’09–’11 (*n* = 1021)	68.15 ± 14.39^a−c,f^	71, 3–97	4.04 ± 3.27^a,b,f^	3.2, 0.3–28.9	5.58 ± 6.58^f^	2.86, 0–57.0	36.6^a,c,f^	3.54 ± 1.97^a,f^	31.2/51.8/14.7/2.3^a,f^
Period’12–’15 (*n* = 1386)	69.3 ± 14.60^a−c,f^	72, 3–94	3.64 ± 3.16^a−d,f^	2.7, 0.3–31.7	6.03 ± 6.90^f^	3.20, 0–36.0	37.1^a,c,f^	3.70 ± 2.04^a^	28.3/53.0/15.2/3.5^a,b^
Period’16–’19 (*n* = 1660)	70.73 ± 13.38^a−e^	74, 13–96	3.41 ± 3.00^a−e^	2.5, 0.2–32.0	6.55 ± 6.83^d,e^	4.22, 0–35.3	38.8^a−e^	3.80 ± 1.95^a,b,d^	25.9/50.5/18.0/2.6^a−d^

Period ‘89–’98, Period ‘99–’01, Period ‘02–’08, Period ‘09–’11, Period 12’–15 and period ‘16–19’ match a, b, c, d, e and f in order. If the diference from the compared period was signifcant (*p* < 0.05), the superscript letter of the period is shown

Abbreviations *AAV* anti-neutrophil cytoplasmic antibody(ANCA)-associated vasculitis, *aGBMD* anti-glomerular basement membrane (GBM) disease, *CRP* C-reactive protein, *CS* grade: clinical severity grade, *GPA* granulomatosis with polyangiitis, *MPA* microscopic polyangiitis, *non-ANCA SV* non-ANCA systemic vasculitis, *n* no., *pGN with cres* primary glomerulonephritis with crescent, *RPGN* rapidly progressive glomerulonephritis, *RLV* renal limited vasculitis, *sCre* serum creatinine, *SLE* systemic lupus erythematosus

### Life prognoses and renal prognoses based on the time of onset for the overall RPGN series

**Fig. 2:**
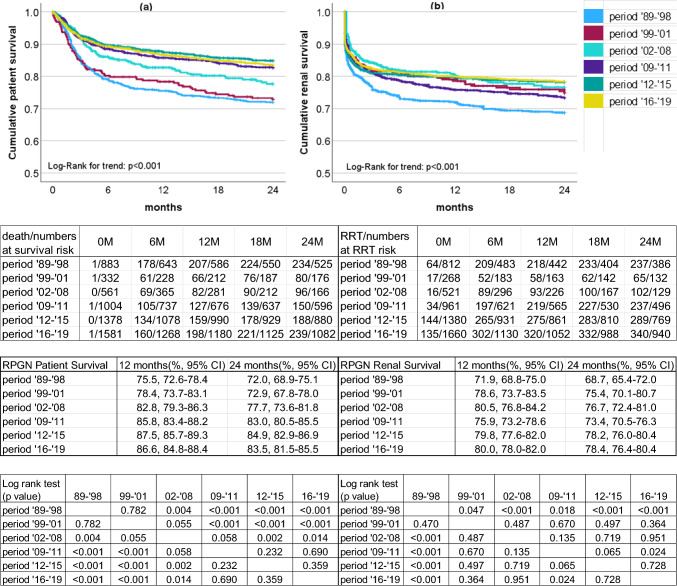
A comparison of the cumulative life and renal prognoses from disease onset to 24 months according to time of onset

The survival curves for the life prognoses and the renal prognoses of the patients from the disease onset to 24 months are shown in Fig. 2a,b. Period ‘16–’19 was the period when the cases were the oldest, but the life and renal prognoses did not deteriorate compared with the best period ‘12–’15.

### Life and renal prognoses depending on the CS grade at onset in overall RPGN

Supplementary Figure S1 depicts the life prognoses in the overall RPGN series over the entire study period by CS grade stratification. As in previous reports, the difference in life prognosis among the four CS grades (I, II, III, IV) were still significant.

### Life and renal prognoses depending on the causative disease

**Fig. 3:**
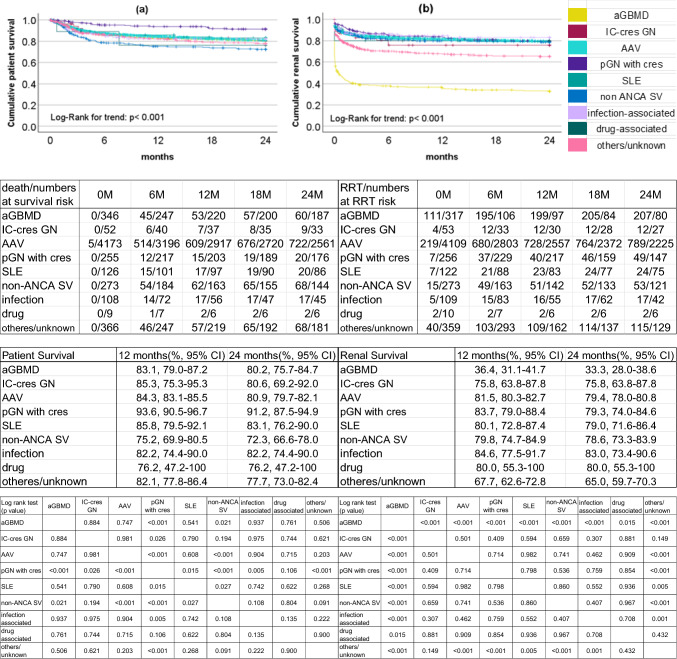
A comparison of the cumulative survival and renal survival from onset to 24 months by causative disease in the overall study period

Figure 3a, b depicts the patients’ life and renal prognoses over the entire period according to the cause of RPGN. The patients with primary glomerulonephritis with crescent formations (pGN with cres) achieved a significantly better life prognosis. The renal prognosis of the patients with anti-GBM disease (aGBMD) was much poorer than those of the patients with any other causative disease.

### Changes over time in the life and renal prognoses of the patients with AAV-RPGN

**Fig. 4:**
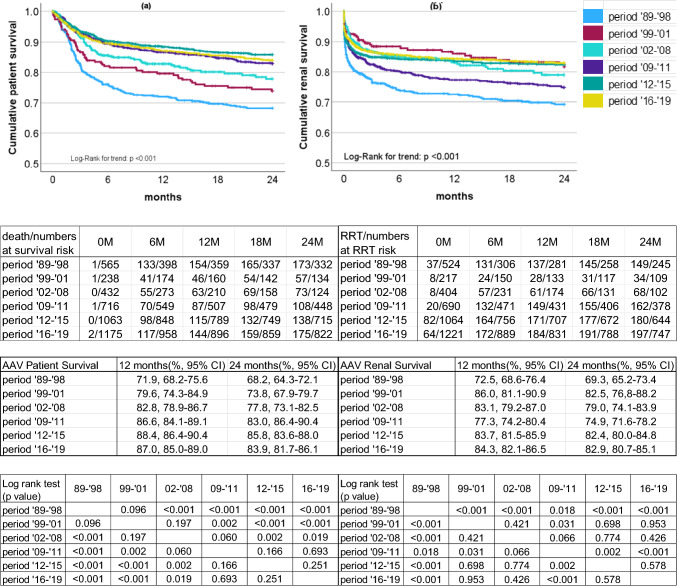
A comparison of the cumulative life and renal prognoses of the patients with AAV-RPGN from onset to 24 months by time of onset

The survival curves for the life and renal prognoses of the patients with AAV- RPGN from disease onset to 24 months are illustrated in Fig. 4a, b. The overall life prognosis of the AAV patients revealed a trend toward improvement over time, although there was no significant difference, the prognosis for those aged 16–19 deteriorated. There was a significant difference in renal outcomes between ‘89–’98 and ‘09–’11, but not between the other time periods. Supplementary Figs. S2–S4 show the comparison of the patients’ life and renal prognoses according to their Cre values at disease onset. Among the patients with low Cre values (< 3 mg/dL), the period ‘89–’98 had the worst life and renal prognoses. In the other two groups (3 ≤ Cre < 6, 6 ≤ Cre), there was no improvement in the renal prognosis.

### Changes in the treatment of AAV-RPGN

The evolution of the treatment content over the entire treatment period is described in Table 2. GC with or without immunosuppressive drugs was used in more than 98% of cases in period ‘12–’15 and period ‘16–’19, even in mild cases(Cre < 3 mg/dL), and was the key drug for treatment against AAV-RPGN in many cases, although there was no significant difference in severe 141 cases (6 ≤ Cre mg/dL) compared to the other periods.

**Table 2:** Tenporal changes in Frequency of treatment for AAV-RPGN

Treatment for AAV-RPGN	Cre, mg/dL	Parcentage of treatment (%)						
		Period ‘89–‘98	Period ‘99–‘01	Period ‘02–‘08	Period ‘09–‘11	Period ‘12–‘15	Period ‘16–‘19	All
GC	< 3	91.5 (150/164)^c−f^	93.4 (85/91)^c,e,f^	82.8 (140/169)^a,b,d−f^	95.5 (321/336)^c−f^	98.3 (590/600)^a−d^	98.2(698/711)^a−d^	95.8(1984/2071)
	3≦, 6 >	93.3 (183/196)^c^	92.2 (71/77)^e–f^	83.4 (141/169)^c−f^	98.3 (232/236)^c^	98.7 (308/312)^b,c^	98.4(305/310)^b,c^	95.4(1240/1300)
	6≦	90.1 (164/182)^e^	87.3 (48/55)^e^	88.2 (75/85)^e^	92.7 (127/137)^e^	98.0 (146/149)^a−d^	95.0(134/141)	92.7(694/749)
SP	< 3	56.7 (93/164)^c^	68.1 (62/91)^e,f^	69.2 (117/169)^a,d−f^	56.5 (190/336)^c^	55.2 (331/600)^b,c^	53.7(382/711)^b,c^	56.7(1175/2071)
	3≦, 6 >	66.8 (131/196)	67.5 (52/77)	66.9 (113/169)	64.4 (152/236)	64.1 (200/312)	69.7(216/310)	66.5(864/1300)
	6≦	75.8 (138/182)^d^	61.8 (34/55)^f^	72.9 (62/85)	64.2 (88/137)^a,e,f^	75.8 (113/149)^d^	76.5(108/141)^b,d^	72.5(543/749)
CY	< 3	47.6 (79/166)^c−f^	49.5 (45/91)^c−f^	31.8 (54/170)^a,b,e^	26.0 (88/339)^a,b^	22.8 (137/601)^a−c^	24.9(177/711)^a,b^	27.9(580/2078)
	3≦, 6 >	50.5 (99/196)^c−f^	50.0 (39/78)^c−f^	29.0 (49/169)^a,e^	23.8 (57/239)^a,b^	19.5 (61/313)^a−c^	21.0(65/310)^a,b^	28.4(370/1305)
	6≦	33.0 (60/182)^d−f^	42.9 (24/56)^d−f^	27.9 (24/86)^d^	16.4 (23/140)^a−c^	21.5 (32/149)^a,b^	22.0(31/141)^a,b^	25.7(194/754)
AZ	< 3	0.6(1/166)^d−f^	1.1(1/91)^d−f^	1.2(2/170)^d−f^	10.6 (36/339)^a−c,e,f^	16.0(96/601)^a−d,f^	25.5(181/711)^a−e^	15.3(317/2078)
	3≦, 6 >	0.6 (1/196)^d−f^	1.3 (1/78)^d−f^	1.2 (2/169)^d−f^	8.8 (21/239)^a−c^	9.6(30/313)^a−c^	13.2(41/310)^a−c^	7.4(96/1305)
	6≦	1.2 (1/182)^d−f^	0 (0/56)^e,f^	1.2 (1/86)^e,f^	7.1(10/140)^a^	9.4(14/149)^a−c^	8.5(12/141)^a−c^	5.0(38/754)
CNI	< 3	1.2 (2/166)^f^	1.1 (1/91)	1.2(2/170)^d,f^	4.7 (16/339)^c^	3.8 (23/601)	5.5(39/711)^a,c^	2.9(61/2078)
	3≦, 6 >	0 (0/196)^d,e^	0 (0/78)	1.8 (3/169)	2.5 (6/239)^a^	4.2 (13/313)^a,f^	1.3(4/310)^e^	1.2(16/1305)
	6≦	1.1 (2/182)	3.6 (2/56)	2.3 (2/86)	2.1 (3/140)	2.0 (3/149)	1.4(2/141)	1.9(14/754)
MZ	< 3	3.6 (6/166)^d−f^	6.6 (6/91)^d^	5.3 (9/170)^d,e^	15.0 (51/339)^a−c,f^	12.6 (76/601)^a,c,f^	4.2(30/711)^a,d,e^	5.1(106/2078)
	3≦, 6 >	3.6 (7/196)^d,e^	1.3 (1/78)^d,e^	5.3 (9/169)	8.4 (20/239)^a,b^	8.6 (27/313)^a,b^	5.2(16/310)	6.1(80/1305)
	6≦	2.2 (4/182)^d^	1.8 (1/56)	3.5 (3/86)	8.6 (12/140)^a,f^	6.7 (10/149)	2.8(4/141)^d^	4.5(34/754)
MMF	< 3				1	0.8 (5/601)	2.5(18/711)	1.2(24/2078)
	3≦, 6 >				1	0.3 (1/313)	1.9(6/310)	0.6(8/1305)
	6≦					0.7 (1/149)	0.7(1/141)	0.3(2/754)
MTX	< 3			1		0.8 (5/601)	1.0(7/710)	0.6(13/2078)
	3≦, 6 >					0 (0/313)	0(0/310)	0.0(0/1305)
	6≦					0 (0/149)	0(0/141)	0.0(0/754)
RIX	< 3					3.8 (23/601)^f^	19.5(139/711)^e^	7.8(162/2078)
	3≦, 6 >					2.9 (9/313)^f^	12.3(38/310)^e^	3.6(47/1305)
	6≦					4.0% (6/149)^f^	14.2(20/141)^e^	3.4(26/754)
PP	< 3	9.2(15/163)^e,f^	5.9 (5/85)	7.4 (12/162)	4.6 (15/326)	3.8 (23/600)^a^	4.5(32/711)^a^	4.9(102/2078)
	3≦, 6 >	12.4(24/194)^e^	11.1 (8/72)	8.0 (13/163)	8.6 (20/233)	6.4 (20/313)^a,f^	13.5(42/310)^e^	9.7(127/1305)
	6≦	20.4 (37/181)^c^	13.0 (7/54)	9.2 (7/76)^a,d−f^	22.7 (30/132)^c^	19.5 (29/149)^c^	23.4(33/141)^c^	19.0(143/754)

Period ‘89–’98, Period ‘99–’01, Period ‘02–’08, Period ‘09–’11, Period ‘12–’15 and Period ‘16–’19 match a, b, c, d, e and f in order. If the diference from the compared period was signifcant (*p* < 0.05), the superscript letter of the period is shown

Abbreviation: *AZ* azathioprine, *CNI* calcineurin inhibitors, *CY* cyclophosphamide, *GC* glucocorticoid, *MMF* mycophenolate mofetil, *MTX* methotrexate, *MZ* mizoribine, *PP* plasmapheresis, *RIX* rituximab, *SP* pulse glucocorticoid

The use of azathioprine increased significantly over time in the mild cases, whereas the use of mizoribine showed a decreasing trend in the same cases. The use of CY had been declining since period ‘02–’08 compared with earlier periods; period ‘16–’19 was not significantly different in CY use from period ‘02–’08 onwards. The use of RIX increased significantly compared to the period ‘12–’15 and increased regardless of the Cre level. The use of plasma exchange increased in the moderately ill patients (Cre 3 to < 6 mg/dL) compared with the period ‘12–’15.

### Initial therapy for AAV-RPGN in 2016–2019

**Fig. 5:**
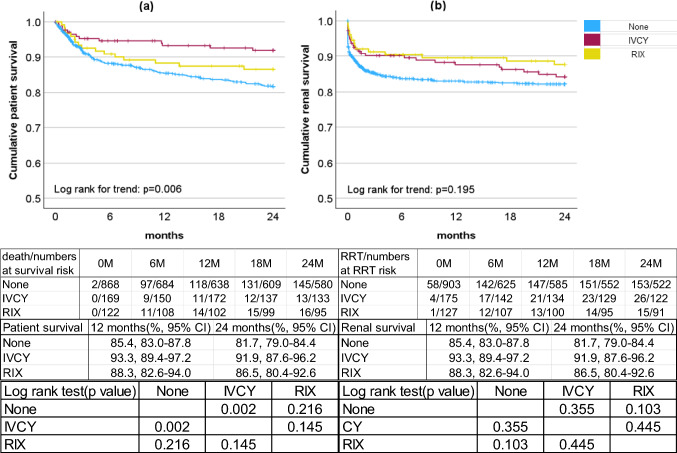
A comparison of cumulative life and renal prognoses of patients with AAV-RPGN from onset to 24 months by initial therapy

Table 3 shows the patient background by initial treatment during the latest survey period ‘16–’19: the patients who received intravenous cyclophosphamide (IVCY) (*n* = 175) or RIX (*n* = 127) tended to be younger and had a higher prevalence of lung lesions than those who did not (None) (*n* = 903). Although there was a trend toward better life and renal prognosis with IVCY or RIX use compared to no immunosuppressant use, the only statistically significant difference was in life prognosis with IVCY use. On the other hand, there was no significant difference in both prognoses between IVCY use and RIX use (Fig. 5a,b). Patient survival and renal survival by Cre at onset and by initial treatment are shown in Supplementary Fig. S5 and S6. For moderate cases (3 ≤ Cre < 6), immunosuppressive drugs tended to have a higher survival rate, although the difference was not significant. While for severe cases (6 ≤ Cre), immunosuppressive drugs improved renal prognosis, with the group of RIX use having the best renal survival rate.

**Table 3:** Clinical characterisitics by initial therapy

	Age	sCre(mg/dL)		CRP(mg/dL)		Lung involvement		Clinical severity classification	
	Mean ± SD	Median, range	Mean ± SD	Median, range	Mean ± SD	Median, range	%	Total score	CS Grade I/II/III/IV(%)
None (*n* = 903)	73.24 ± 11.02^b,c^	75, 13–94	3.18 ± 2.64	2.35, 0.35–25.70	6.29 ± 6.61^b,c^	3.80, 0–32.96	42.4^b,c^	3.89 ± 1.86^b^	23.2/54.7/16.8/2.7
IVCY (*n* = 175)	70.65 ± 9.94^a^	72, 42–96	2.97 ± 2.81	2.05, 0.45–20.33	8.03 ± 6.85^a^	7.58, 0.01–29.90	57.1^a^	4.17 ± 1.75^a^	18.9/52.0/24.6/1.7
RIX (*n* = 127)	71.02 ± 11.47^a^	73, 21–88	2.73 ± 1.97	2.26, 0.54–9.06	7.83 ± 7.14^a^	7.115, 0.01–25.8	55.9^a^	4.06 ± 2.01	21.3/49.6/25.2/3.1

None, IVCY and RIX match a, b and c in order. If the diference from the compared period was signifcant (*p* < 0.05), the superscript letter of the period is shown

Abbreviations *None* no immunosuppressive drug, *IVCY* intravenous cyclophosphamide, *RIX* rituximab

### Changes over time in the life and renal prognoses of the RPGN patients with aGBMD-RPGN

The survival curves for the life prognosis and renal prognosis of the patients with aGBMD-RPGN from onset to 24 months are presented in Supplementary Fig. S7. The aGBMD-RPGN group continued to have the same grim renal prognosis as before, but improved in the current period compared with period ‘12–’15, which was the worst renal prognosis.

### The causes of death in the total RPGN series

Table 4 shows the changes in the respective causes of death among the patients with RPGN. For cause of death, multiple causes could be selected because of the questionnaire method and the paper-based entry system. Death from infection during the latest period was the lowest among all six of the periods, and similarly, death from infection was the lowest in the AAV patients; both of these showed a trend toward improvement over time.

**Table 4:** Cause of death

	Infection	Interstital pnuemonitis	Pulmonary hemorrhage	Heart failure	Myocardial infarction	Gastrointestinal bleeding, perforation	Brain hemorrhage	Others/Unknown
*antiGBMD*								
Period’89-’98 (*n* = 13)^#^	76.9 (10)^b,d,e^	15.4 (2)	7.7 (1)	7.7 (1)	7.7 (1)	15.4 (2)	7.7 (1)	0 (0)^d,e^
Period’99-’01 (*n* = 5)	0 (0)^a,c^	0 (0)	20.0 (1)	0 (0)	0 (0)	40.0 (2)	0 (0)	40.0 (2)
Period’02-’08 (*n* = 10)^#^	70.0 (7)^b,d^	20.0 (2)	0 (0)	20.0 (2)	10.0 (1)	10.0 (1)	10.0 (1)	20.0 (2)^d^
Period’09-’11 (*n* = 5)	0 (0)^a,c^	0 (0)	0 (0)	0 (0)	0 (0)	0 (0)	0 (0)	100 (5)^a,c,e,f^
Period’12-’15 (*n* = 14)	28.6 (4)^a^	0 (0)	14.3 (2)	0 (0)	0 (0)	14.3 (2)	7.1 (1)	35.7 (5)^a,d^
Period’16-’19 (*n* = 15)^#^	40.0 (6)	0 (0)	13.3 (2)	6.6 (1)	6.6 (1)	0 (0)	6.6 (1)	33.3 (5)^d^
*Overall AAV*								
Period’89-’98 (*n* = 172)^#^	57.6 (99)^e,f^	14.0 (24)^d^	17.4 (30)^d−f^	7.6 (13)	0.6 (1)	13.4 (23)^e^	5.2 (9)	20.3 (35)^c,e,f^
Period’99-’01 (*n* = 57)^#^	43.9 (25)	19.3 (11)^d−f^	8.8 (4)	8.8 (5)	5.3 (3)	14.0 (8)^e^	7.0 (4)	29.8 (17)
Period’02-’08 (*n* = 73)^#^	52.4 (39)^f^	20.5 (15)^d−f^	11.0 (8)	1.4 (1)	0 (0)	5.5 (4)	2.7 (2)	37.0 (27)^a,d^
Period’09-’11 (*n* = 111)	52.2 (58)^f^	6.3 (7)^a−c^	4.5 (5)^a^	1.8 (2)	0.9 (1)	8.1 (9)	5.4 (6)	20.7 (23)^c,f^
Period’12-’15 (*n* = 139)^#^	40.3 (56)^a^	7.2 (10)^b,c^	7.2 (10)^a^	6.5 (9)	0.7 (1)	2.9 (4)^a,b^	6.5 (9)	38.8 (54)^a,d^
Period’16-’19 (*n* = 175)^#^	37.7 (66)^a,c,d^	8.6 (15)^b^	7.4 (13)	4.6 (8)	1.7 (3)	6.9 (12)	2.3 (4)	41.1 (72)^a,d^
*MPA(+ RLV)*								
Period’89-’98 (*n* = 149)^#^	59.1 (88)^e,f^	15.4 (12)^d,e^	19.5 (29)^d−f^	8.1 (12)	0.7 (1)	12.1 (18)^e^	6.0 (9)	18.8 (28)^c,e,f^
Period’99-’01 (*n* = 47)^#^	48.9 (23)	23.4 (11)^d−f^	8.5 (4)	10.6 (5)^c^	4.2 (2)	14.9 (7)^e^	6.3 (3)	27.7 (13)
Period’02-’08 (n = 59)^#^	54.2 (32)	20.3 (12)^d,e^	11.9 (7)	0 (0)^b^	0 (0)	5.1 (3)	3.4 (2)	35.6 (21)^a^
Period’09-’11 (*n* = 93)	50.5 (47)	5.4 (5)^a−c^	5.4 (5)^a^	2.1 (2)	0 (0)	9.7 (9)	6.5 (6)	20.4 (19)^f^
Period’12-’15 (*n* = 137)^#^	39.4 (54)^a^	7.3 (10)^a−c^	6.6 (9)^a^	6.6 (9)	0.7 (1)	2.9 (4)^a,b^	6.6 (9)	38.6 (53)^a^
Period’16-’19 (*n* = 171)^#^	37.4 (64)^a^	8.8 (15)^b^	7.0 (12)^a^	4.7 (8)	1.8 (3)	7.0 (12)	2.3 (4)	41.5 (71)^a,d^
*GPA*								
Period’89-’98 (*n* = 5)^#^	40.0 (2)	0 (0)	0 (0)	0 (0)	0 (0)	60.0 (3)	0 (0)	20.0 (1)
Period’99-’01 (*n* = 4)	25.0 (1)	0 (0)	0 (0)	0 (0)	0 (0)	25.0 (1)	25.0 (1)	25.0 (1)
Period’02-’08 (*n* = 4)^#^	50.0 (2)	25.0 (1)	0 (0)	0 (0)	0 (0)	0 (0)	0 (0)	50.0 (2)
Period’09-’11 (*n* = 3)	100.0 (3)	0 (0)	0 (0)	0 (0)	0 (0)	0 (0)	0 (0)	0 (0)
Period’12-’15 (*n* = 3)^#^	66.7 (2)	0 (0)	33.3 (1)	0 (0)	0 (0)	0 (0)	0 (0)	33.3 (1)
Period’16-’19 (*n* = 4)	50.0 (2)	0 (0)	25.0 (1)	0 (0)	0 (0)	0 (0)	0 (0)	25.0 (1)
*Total of overall RPGN*								
Period’89-’98 (*n* = 234)^#^	59.4 (139)^b,e,f^	12.4 (29)^d−f^	14.5 (34)^d−f^	7.7 (18)^d^	0.9 (2)	11.5 (27)^b,e,f^	6.4 (15)	19.7 (46)^c,e,f^
Period’99-’01 (*n* = 80)^#^	45.0 (36)^a^	17.5 (14)^d−f^	8.8 (7)	11.3 (9)^d,f^	3.8 (3)	20.0 (16)^a,c−f^	6.3 (5)	30.0 (24)^e,f^
Period’02-’08 (*n * = 96)^#^	55.2 (53)^e,f^	17.7 (17)^d−f^	8.3 (8)	4.2 (4)	1.0 (1)	6.25 (6)	3.1 (3)	35.4 (34)^a^
Period’09-’11 (*n * = 155)	50.9 (79)^e,f^	4.5 (7)^a−c^	3.9 (6)^a^	1.9 (3)^a,b^	0.6 (1)	7.1 (11)^b^	3.9 (6)	27.1 (42)^e,f^
Period’12-’15 (*n * = 189)^#^	37.0 (70)^a,c,d^	5.3 (10)^a−c^	6.9 (13)^a^	1.9 (3)	0.5 (1)	4.2 (8)^a,b^	5.8 (11)	43.4 (82)^a,b,d^
Period’16-’19 (*n * = 239)^#^	35.1 (84)^a,c,d^	6.7 (16)^a−c^	6.7 (16)^a^	5.3 (10)	1.7 (4)	5.9 (14)^a,b^	2.9 (7)	45.2 (108)^a,b,d^

Period ‘89–’98, Period ‘99–’01, Period ‘02–’08, Period ‘09–’11, Period ‘12–’15 a*n *d Period ‘16–’19 match a, b, c, d, e and f in order. If the diference from the compared Period was signifcant (*p* < 0.05), the superscript letter of the period is shown. Brain hemorrhage includes cerebral hemorrhage, cerebellar hemorrhage and subarachnoid hemorrhage.The clinician judged the cause of death to be a composite cause of death in the questionnaire, and # was given for periods when more than one cause of death was selected

Abbreviation *AAV* anti-neutrophil cytoplasmic antibody(ANCA)-associated vasculitis, *aGBMD* anti-glomerular basement membrane (GBM) disease, *GPA* granulomatosis with polyangiitis, *MPA* microscopic polyangiitis, *RPGN* rapidly progressive glomerulonephritis, *RLV* renal limited vasculitis

## Discussion

We have conducted an ongoing epidemiological study of patients with RPGN in Japan since 1989, with 1,660 new cases added to the present study, bringing the cumulative total of cases registered in 1989–2019 to 5,839. No other report has continuously evaluated the life expectancy, renal prognosis, and changes in treatment methods over such a long course. The earlier use of this study’s survey revealed that the life expectancy of RPGN patients in Japan has improved together with a reduction of the intensity of treatment from conventional therapy since 2002, as RPGN is characterized by a high proportion of elderly patients and MPO-ANCA-positive patients, and approx. one-half of all deaths were caused by infections. Previous investigations have also shown improvements in renal prognosis [1], which were attributed to an increase in the number of mild cases (Cre < 3) contributing to improved renal prognoses with the endpoint of the initiation of renal replacement therapy.

In previous surveys, the RPGN patients’ life expectancy tended to improve with each new survey, though it remained at the same level in the current survey. The patients’ renal prognoses also remained at the same level as in the previous study [1]; we observed a similar trend when the analysis was restricted to AAV-RPGN cases, with a similar improvement in renal prognosis observed in mild cases (Cre < 3 mg/dL) as reported in the previous study, whereas the present moderate and severe cases (≥ 3 Cre mg/dL) had similar renal prognoses. The reason why the life and renal prognosis did not deteriorate in all cases of RPGN overall and AAV-RPGN in the present study, despite the aging of the population, may be due to the fact that many cases were diagnosed early and the Cre at onset was reduced. The following discussion regarding the evolution and transition of the renal prognosis of AAV-RPGN patients is discussed below.

The prognosis of individuals with AAV in other countries and its association with immunosuppressive therapy should be considered. There have been several reports on the prognosis of AAV-RPGN patients. For example, a study of 181 patients diagnosed in the period 1979–2009 in the Netherlands (mean age 61.4 yrs, MPO-ANCA 50.8%, mean Cre 3.56 mg/dL) obtained a 1-year survival rate of 77.2% and a 5-year survival rate of 65.8%, with 1-year and 5-year renal survival rates of 66.7% and 53.5%, respectively; the study’s comparisons demonstrated a trend toward improvement over time [12]. The 1- and 5-year survival rates reported from Spain (mean patient age 60.2 yrs, MPO-ANCA 64.6%, mean Cre 3.89 mg/dL) were 80.9% and 72.1%, respectively, with a 1-year renal survival rates at 100%, 79%, and 63% in the low-, intermediate-, and high-risk groups in a pathologically evaluated risk assessment, respectively, and 5-year renal survival rates at 100%, 77% and 53%, respectively [13]. Other reports from China (median age 60 yrs, MPO-ANCA 87.2%, eGFR 17.5 mL/min/1.73 m^2^, median follow-up 36 months) and France (median age 67 yrs, MPO-ANCA 71%, eGFR 20 mL/min, median follow-up 59 months) showed renal death rates during the follow-up period at 40.4% and 26%, respectively [14, 15]. In those studies, the renal prognosis in the high-risk population classified by the Renal Risk Score was around ≤ 50% at 2–5 years, which is similar to the renal prognosis in severe cases in Japan.

Elderly individuals with AAV-RPGN have a poor life and renal prognoses [16, 17], and an increased age at onset may be a factor in the lack of improvement in life and renal prognoses. As in previous investigations performed in Japan, the use of CY as described in the present survey was lower compared to reports from other countries. The use of CY may have been low in earlier Japanese studies due to the high number of elderly patients in Japan and the countermeasures used against infectious disease deaths in the early days of treatment. CY has been reported to have a high remission rate in initial treatment, and it cannot be denied that its low use in Japan is associated with a lack of improvement in renal prognosis in severe cases. Improved survival has also been reported in elderly patients treated with CY compared to that of elderly patients treated with glucocorticoids alone [18, 19], which may be related to a reduced glucocorticoid exposure with immunosuppressive drug combinations [9].

RIX alone or in combination with CY has shown efficacy that is comparable to that of CY [9, 10], and national and international treatment guidelines therefore recommend the combination of CY or RIX. The use of RIX was first covered by Japan’s health insurance in 2012; the increased use of RIX since 2017 (when it was included in Japanese treatment guidelines) resulted in a significant change in the nationwide survey’s results. However, as the present survey included the period prior to the inclusion of RIX in the Japanese treatment guidelines, it is expected that the frequency of RIX use will further increase in future. Although the frequency of CY use has remained unchanged, that of RIX use has increased, indicating an increase in the frequency of a concomitant use of immunosuppressive drugs or biologicals in initial treatment and a change in treatment selection trends in Japan. Although it is difficult to exclude bias and it is not possible to argue the superiority or inferiority of treatment methods as our present investigation was not an intervention study, we speculate that a trend toward better renal outcomes in patients with severe renal dysfunction treated with immunosuppressive drugs or biologicals in combination with an initial treatment may contribute to improved renal outcomes in future.

We next discuss the indications and implementation of plasma exchange (PLEX), which can be effective for patients with severe renal impairment and is an option for patients with severe renal failure and a low risk of infection, although there is a trade-off between improved renal outcome and risk of infection [20, 21]. Although PLEX as a treatment for AAV-RPGN has been covered by insurance in Japan since 2018 and is therefore limited to part of the present study period, insurance coverage guarantees sufficient treatment volume and frequency. The increasing use of PLEX that we observed for moderate cases (3 ≤ Cre < 6) should be continuously investigated to determine whether it contributes to improved renal prognosis.

On the other hand, the lack of improvement in renal prognosis in moderate to severe cases can be partly attributed to the fact that renal replacement therapy becomes inevitable at the stage of referral to a nephrologist. In the present study, it was also observed that renal replacement therapy was initiated at the start of treatment, particularly in severe cases. This underscores the importance of early specialist intervention in situations where renal function is progressively declining and recovery becomes more difficult. Additionally, this may be influenced by the prevailing treatment approach in elderly patients, particularly in moderate to severe cases, where the priority is to avoid infectious death rather than renal death. This treatment philosophy, which has been a long-standing practice in Japan, often necessitates a more cautious approach in the management of elderly patients. Looking ahead, it is essential to strengthen the cooperation between primary care physicians and nephrologists in order to establish a system that enables earlier intervention. Furthermore, exploring treatment strategies that include the use of appropriate immunosuppressive drugs is crucial for improving renal prognosis.

The renal prognosis of patients with anti-GBMD has remained unimproved. A Danish study of the renal prognosis of anti-GBMD patients obtained data that are similar to our present findings, with a 5-year mortality rate at 26.8% and a 62.9% rate of renal replacement therapy at 1 year [22]. Although the patient age at disease onset in the present study is older than that in our previous study [1] and similar to that in the Danish study, the lack of deterioration in life and renal prognoses may be seen as an improvement in the management of anti-GBMD, despite the expected increase in mortality and decline in renal function associated with aging. The absence of worsening life and renal prognoses may be associated with an improved management of anti-GBMD. The renal prognosis of patients who become dependent on dialysis within one month of diagnosis is worse than that of patients who do not become dependent on dialysis, and thus, the early diagnosis and therapeutic interventions appropriate for anti-GBMD remain a challenge.

The strengths of our study are as follows: (*i*) it is a continuous survey, allowing changes over time in Japan to be recorded, and (*ii*) it is an epidemiological survey conducted on a scale not seen elsewhere, with responses from approx. 40% of the renal care centers in Japan. However, this was also a backward-looking study and it does not have strong evidence on the superiority of treatments.

Treatment methods continue to change, with the use of biological agents increasing and the number of cases treated with GC alone decreasing. With further promotion of RIX and the introduction of a novel treatment for AAV, i.e., avacopan, after this study’s time period, continued investigations are needed to determine whether further improvements in these patients’ life and renal outcomes can be achieved.

## Conclusion

Despite an increasingly older age of onset, both life and renal prognoses for new-onset AAV-RPGN from 2016 to 2019 remain comparable to the best in previous surveys, due to the impact of constant improvements in early diagnosis and changes in treatment.

## Supplementary Information

Below is the link to the electronic supplementary material.

Supplementary file1 (DOCX 50 KB)

Supplementary file2 (DOCX 95 KB)

Supplementary file3 (DOCX 106 KB)

Supplementary file4 (DOCX 89 KB)

Supplementary file5 (DOCX 56 KB)

Supplementary file6 (DOCX 64 KB)

Supplementary file7 (DOCX 82 KB)
