# Supplementary material for: Temporal changes of the life and renal prognoses of patients with rapidly progressive glomerulonephritis in Japan, 1989–2019
Source: Clin Exp Nephrol. 2025 Mar 25;29(7):937–52. doi: 10.1007/s10157-025-02643-6 (PMC12204914; doi:10.1007/s10157-025-02643-6)
Supplement: Supplementary file 1 — Supplementary file1 (DOCX 50 KB) [file 10157_2025_2643_MOESM1_ESM.docx]

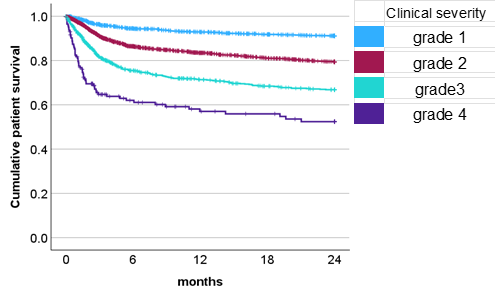

Suppl. Fig. S1. A comparison of the cumulative survival survival rates from disease onset to 24 months depending on the clinical severity (CS) grade in the overall study period.

Nakajima K et al. Supplemental figure 1
