# Supplementary material for: Temporal changes of the life and renal prognoses of patients with rapidly progressive glomerulonephritis in Japan, 1989–2019
Source: Clin Exp Nephrol. 2025 Mar 25;29(7):937–52. doi: 10.1007/s10157-025-02643-6 (PMC12204914; doi:10.1007/s10157-025-02643-6)
Supplement: Supplementary file 3 — Supplementary file3 (DOCX 106 KB) [file 10157_2025_2643_MOESM3_ESM.docx]

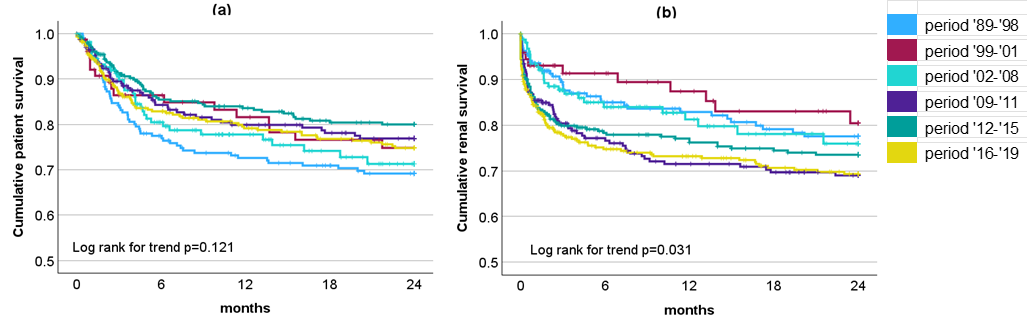

Suppl. Fig. S3. A comparison of the cumulative life and renal prognoses of the patients with moderate AAV-RPGN (3≤Cre<6 mg/dL) from onset to 24 months by time of onset.

Nakajima K et al. Supplemental figure 3
