# Supplementary material for: Temporal changes of the life and renal prognoses of patients with rapidly progressive glomerulonephritis in Japan, 1989–2019
Source: Clin Exp Nephrol. 2025 Mar 25;29(7):937–52. doi: 10.1007/s10157-025-02643-6 (PMC12204914; doi:10.1007/s10157-025-02643-6)
Supplement: Supplementary file 6 — Supplementary file6 (DOCX 64 KB) [file 10157_2025_2643_MOESM6_ESM.docx]

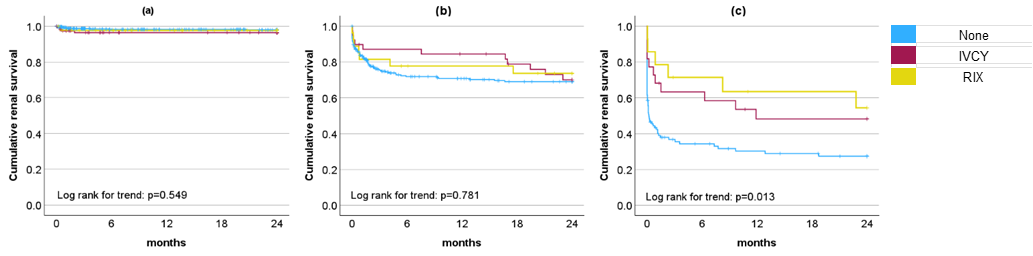

Suppl. Fig. S6. A comparison of cumulative renal prognosis of patients with AAV-RPGN stratified by Cre at onset (a:Cre <3, b:3≤Cre<6, c:6≤Cre) from onset to 24 months by initial therapy

Nakajima K et al. Supplemental figure6
